# No benefit in biomarkers assessing muscle damage for minimally invasive anterior over SPAIRE approach in hemiarthroplasty: a subgroup analysis with 100 patients from a randomized controlled trial

**DOI:** 10.1302/2633-1462.610.BJO-2025-0027.R1

**Published:** 2025-10-10

**Authors:** Stein H. Ugland, Oystein T. Fagerberg, Knut E. Mjaaland, Terje O. Ugland, Glen Haugeberg, Are H. Pripp, Lars Nordsletten

**Affiliations:** 1 Department of Orthopaedics, Sorlandet Hospital, Kristiansand, Norway; 2 Institute of Clinical Medicine, University of Oslo, Oslo, Norway; 3 Department of Orthopaedics, Sorlandet Hospital, Arendal, Norway; 4 Department of Neurosciences, Rheumatology Division, Norwegian University of Science and Technology, Trondheim, Norway; 5 Division of Rheumatology, Department of Internal Medicine, Sorlandet Hospital, Kristiansand, Norway; 6 Oslo University Hospital and Oslo Metropolitan University, Oslo, Norway; 7 Division of Orthopaedic Surgery, Oslo University Hospital, Oslo, Norway

**Keywords:** Hip fracture, SPAIRE approach, hemiarthroplasty, muscle damage, biomarkers, CRP, randomized controlled trial, inflammation, clinical outcomes, femoral neck fractures, obturator internus muscles, piriformis

## Abstract

**Aims:**

Muscle damage and inflammation after hemiarthroplasty (HA) for femoral neck fracture (FNF) could affect time to mobilization. Early mobilization is key in enhanced recovery and fast-track care systems. We have compared muscle damage and inflammation using creatine kinase (CK) and CRP as biomarkers in FNF patients operated on with the direct anterior (DA) and the sparing piriformis and obturator internus, repairing externus (SPAIRE) approach.

**Methods:**

From January 2022, 158 eligible patients with a dislocated FNF were included in a randomized controlled trial comparing the approaches (n = 158). Hypothesis and planned statistical tests were pre-specified in this sub-group analysis and 100 patients were tested for CK, CRP, and haemoglobin (Hb) levels during hospital admission.

**Results:**

Mean difference in CK between groups was, on postoperative day one, 45 u/l (95% CI -22 to 151, p = 0.290) and day two, 66 U/l (95% CI -42 to 185, p = 0.19). Mean difference in CRP was 3 mg/l (95% CI -23 to 19, p = 0.933) and Hb, 0.3 g/dl (95% CI -0.2 to 0.5, p = 0.388) on day two postoperatively. No correlation was found between CK/CRP and Timed Up and Go test and Harris Hip Score.

**Conclusion:**

There were no differences in CK and CRP changes between the groups on day one and two after surgery. No correlation was found between CK and clinical outcomes in FNF patients operated with SPAIRE and the DA approach.

Cite this article: *Bone Jt Open* 2025;6(10):1232–1238.

## Introduction

Femoral neck fracture (FNF) is a major trauma primarily affecting elderly people. Significant effort has been made in optimizing clinical results; however, one-year mortality rates of 15% to 30% are still reported.^1,2^ Surgery-specific and general complications remain elevated with infections, dislocations, anaemia, respiratory- and urinary tract infections being main causes of readmission to hospital after surgery.^3-5^ Continuous work in enhanced recovery through fast-track care systems attempts to improve the clinical outcome and reduce secondary medical complications. The effect of fast-track care systems on reoperation rates and mortality are questionable.^6,7^ However, studies investigating the effect of early mobilization of FNF patients operated with hemiarthroplasty (HA) report increased recovery rates.^8,9^ Several factors affect the ability to mobilize patients early. Surgical approach has received much attention during the last decade due to several studies reporting improved outcomes when muscle sparing approaches are compared to conventional approaches.^10,11^ Some of these studies have reported less pain and increased mobility in muscle sparing approaches compared to posterior approach (PA) or direct lateral (DL) approach.^12^ Four distinct approaches to the hip joint are used regularly and these approaches have different effects on muscular damage and inflammation. The DL approach, described by Hardinge,^13^ has dominated in HA due to a low dislocation risk but is increasingly unpopular in total hip arthroplasty (THA) due to increased risk of trochanteric pain and limping due to damaged hip abductors.^14,15^ The direct anterior (DA) and the modified anterolateral (AL) approaches have gained popularity due to good clinical results and their muscle-sparing concept.^12,16^ The PA is widely used in THA but is associated with an increased dislocation risk in HA.^17-19^ The approaches have advantages and disadvantages and there is no consensus on which surgical approach generates the best results. The 2023 annual report from the Norwegian Arthroplasty Register clearly shows that Norwegian orthopaedic surgeons prefer the PA in THA and DL approach in HA.^17^ This often results in hospitals and surgeons having two surgical approaches in daily use, one for each indication. For quality and logistical reasons, we became interested in the sparing piriformis and obturator internus, repairing externus (SPAIRE) approach,^20^ possibly being a solution for HA.

The SPAIRE approach, a modification of the PA, is becoming increasingly popular due to indications of reduced dislocation risk in HA.^21,22^ The approach leaves the short external rotators attached.^20^ After being introduced in 2019 17% of HAs in Norway were in 2023 implanted using the SPAIRE approach,^17^ even though evidence supporting its use is relatively scarce. Charity et al^23^ have reported promising results in a single-centre cohort of 285 FNF patients operated with the SPAIRE approach. The odds of returning to pre-injury level was higher and the 120-day survival rate improved in the SPAIRE group compared to a matched cohort of FNF patients operated with the DL approach.^23^ Fewer muscles are detached using the SPAIRE approach and this could affect mobilization through positive effects on muscle damage and inflammation.

The use of serum markers of muscle damage and inflammation may offer an objective measure of the invasiveness of the surgical approach. CK is known to directly correlate to muscle damage.^24^ The postoperative rise in serum CK indicates the level of muscle damage and the rise in CRP indicates the level of inflammation.^25,26^ However, published reports diverge regarding changes in CK after DA approach.^25,27^ To our knowledge, muscle damage and inflammation has not been investigated using the SPAIRE approach. We designed a randomized controlled trial (RCT) comparing the SPAIRE approach and the DA approach in HA for FNF and planned a sub-group analysis investigating CK and CRP as biomarkers of muscle damage and inflammation.

## Methods

### Study design and population

A RCT was conducted between January 2022 and July 2024 comparing the DA and the SPAIRE approaches in dislocated FNF. Included patients were operated on at Sorlandet Hospital Kristiansand and Sorlandet Hospital Arendal, Norway, with cemented HA according to randomization. Primary outcome measure was Harris Hip Score (HHS)^28^ at three and 12 months. This sub-group analysis was predefined in the study protocol and consisted of two parallel intervention groups (one:one ratio). A total of 158 patients were included in the main RCT. Overall, 85 patients were, according to sample size calculation, enrolled in this sub-group analysis. To account for missing values, 100 patients were consecutively included from study start. Inclusion criteria were ambulatory patients aaged between 70 and 90 years without dementia prior to sustaining the dislocated FNF. Patients with dementia, fracture in pathological bones, sepsis or local infection and retained hardware interfering with planned HA were not eligible.

This sub-group analysis is part of a larger RCT. A paper investigating changes in bone mineral density between the groups has been published,^29^ and the main functional outcomes will be presented in a future paper. Patient characteristics are listed in Table I.

**Table I.** Patient characteristics.

Variable	DA (n = 52)	SPAIRE (n = 48)
Mean age (SD)	80 (5.8)	80 (4.7)
**Sex, n (%)**		
Female	28 (54)	32 (68)
Male	24 (45)	16 (32)
**ASA grade, n (%)^30^**		
I/II	20 (38)	15 (31)
III/IV	32 (62)	33 (69)
Mean BMI, kg/m^2^ (SD)	24 (3.5)	24,1 (3.4)
Mean HHS (SD)	88 (13)	90 (12)
**Time from fracture to surgery, hrs**		
0 to 12	6	8
12 to 24	25	19
24 to 48	16	10
> 48	6	10

ASA, American Society of Anesthesiologists (36); DA, direct anterior; HHS, Harris Hip Score preoperatively; SPAIRE, sparing pirirformis and obturator internus repair externus.

Eligible patients were included after informed consent by the surgeon on call (SHU, OTF, KEM). The same physician randomized for surgery with either of the approaches according to the randomization list. Randomization was done using sealed and light proof envelopes. A statistician (AHP) created the randomization list stratified by study site. Allocation was based on random block size^2-10^ containing even numbers within each block and even study group numbers. All participants were analyzed according to their allocation at randomization.

### Surgery and implants

Patients were, after randomization, operated by surgeons familiar with the approaches. After obtaining informed consent from patients or proxy, patients were operated within 48 hours with the C-STEM AMT (DePuy Synthes, USA), a highly polished triple tapered component. Palacos R + G (Heraeus, Germany) cement was used together with a 28 mm Articuleze femoral head and the self-centering Bi-polar Head (both from DePuy Synthes, USA). Patients in the DA group were operated in supine position without traction with blunt dissection between the sartorius and tensor facia latae muscles. The SPAIRE approach was performed in the lateral decubitus position with detachment of obturator externus and proximal parts of the quadratus femoris muscles and repair of capsule and obturator externus with non-absorbable PremiCron (B. Braun, Germany) suture.

Spinal anaesthesia, four doses of cefazolin (2 gm every three hours) and 40 mg of low-dose heparin (enoxaparin) were routinely given. In addition, 1 gm of tranexamic acid was administered unless contraindicated. Patients were attempted to be mobilized on the day of surgery without weightbearing restrictions. Walking aids were provided, and physiotherapy commenced on day 1 postoperatively. Both treatment groups received identical postoperative therapy, including the same standard analgetic protocol. Patients were discharged from the hospital with a physiotherapy report including standardized recommendations to outpatient physiotherapists. Study personnel obtaining outcome measures from included patients were blinded by not having access to study papers and by examining them with clothing covering the affected hip. Patients were informed of the randomization result if specific interest was expressed.

### Biomarkers

CK is mainly found in skeletal muscle and is a biomarker of muscle damage.^24^ There has been reported high correlation between CK levels and the length and depth of the surgical dissection in spinal surgery^31^ and some THA studies have indicated a correlation between duration of surgery and postoperative CK levels.^32,33^ The results from studies comparing differences in CK and CRP in THA and HA patients differ, reporting both increased and decreased CK levels when comparing minimally invasive DA to more conventional PA or DL approaches.^25,27^ The measurement of CK offers an objective method to determine the relative invasiveness of a surgical procedure. The reference range is 30 u/l to 135 u/l for females and 55 u/l to 170 u/l for males, with variations due to age, muscle mass, and race. CRP, normally < 5 mg/l, is a commonly used biomarker of infection and inflammation and reacts to, among others, surgical trauma. We analyzed total serum CK by an ultraviolet kinetic method utilizing the Cobas6000 analyzer until September 2022 and thereafter using Cobas Pro (Roche Diagnostics, Switzerland). The analyzer was, according to prior investment plans in Hospital 1, replaced in September 2022. Preoperative and postoperative days one and two samples were obtained together with serum markers for haemoglobin (Hb) and CRP. Baseline was defined as pre-surgery and change in absolute CK (U/ml) and CRP (mg/l) was measured at 24 and 48 hours post-surgery. Hb (g/dl) was measured at admission and 24 hours post-surgery.

### Statistical analysis

The aim of this study was to determine possible differences in CK and CRP as biomarkers of muscle damage and inflammation. Power calculation was carried out estimating a low positive correlation between changes in biomarkers and functional outcomes. A sample size of 85 patients was required to detect an expected correlation coefficient of 0.3 with 80% statistical power and 5% significance level. The groups were analyzed by intention to treat principle. Histograms, Q-Q plots and the Shapiro-Wilk test was used to investigate for normality. A independent-samples *t*-test was used to analyze normally distributed variables, and the Mann-Whitney U test was used for non-normally distributed variables. A paired *t*-test was conducted to compare changes in CK, CRP, and Hb from baseline to follow-up days one and two. Due to only two repeated measurements on days one and two we did not perform adjustments for multiple tests. To exclude possible multiple comparisons issues Bonferroni correction was planned if significant differences occurred and the association between CK/CRP and continuous outcomes was assessed with Pearson correlation coefficient. A p-value ≤ 0.05 was considered statistically significant. SPSS Statistics for Windows v. 21 (IBM, USA) was used for statistical analysis.

### Ethics, registration, funding, and conflicts of interest

Regional Committee for Medical and Health Research Ethics, South-East Norway, approved the trial on 15 March 2021 (153700). The study was registered at Clinical Trials.gov (NCT04900506) and all patients were included after signed consent according to the Declaration of Helsinki.^34^ The study was reported according to the principles of the CONSORT statement (Supplementary Material).

## Results

**Fig. 1:**
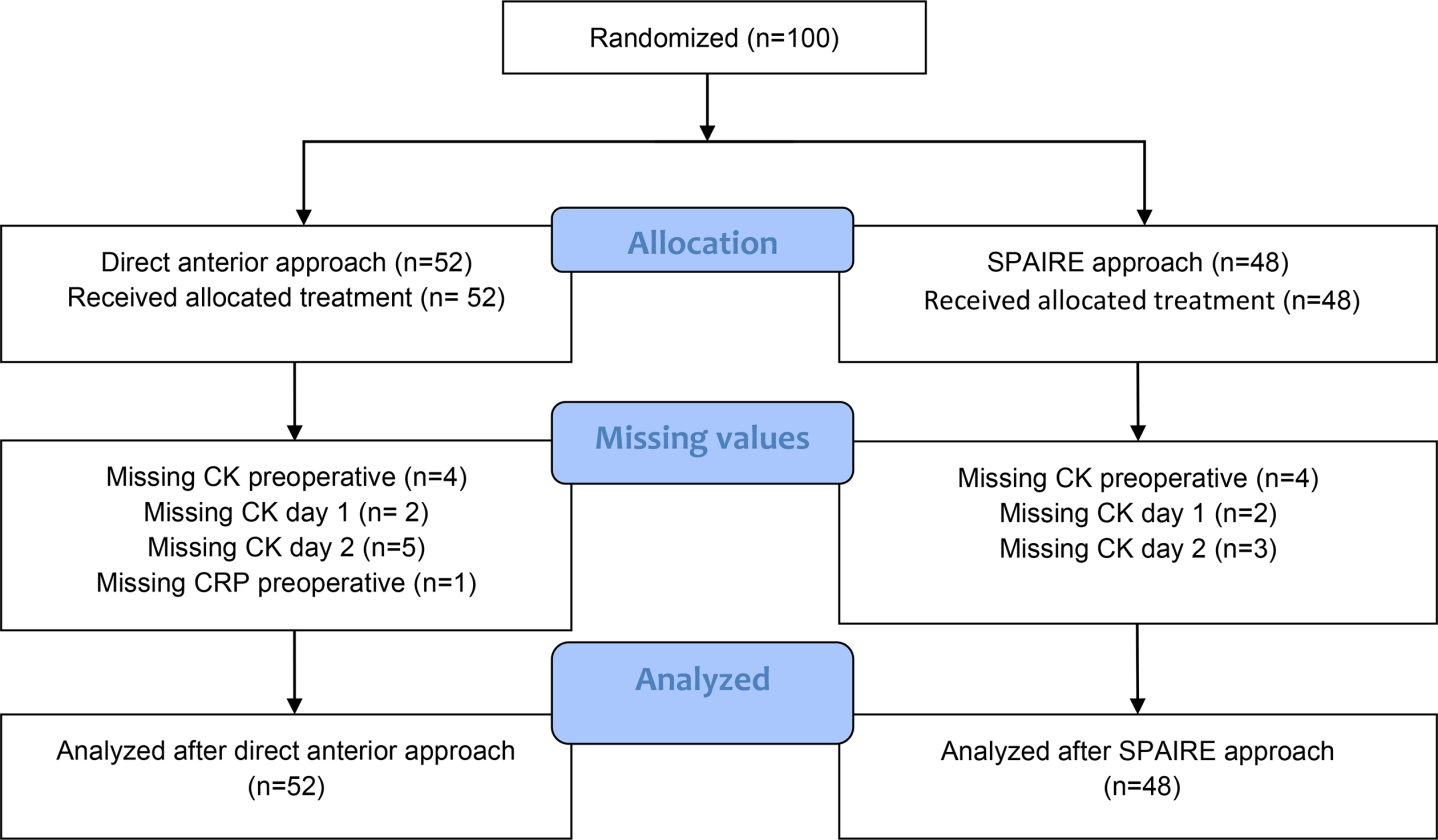
Flowchart. CK, creatine kinase; SPAIRE, sparing piriformis and obturator internus, repairing externus.

In total, 100 patients were consecutively included in this pre-planned sub-group analysis; 86 patients had complete CK tests, 14 patients had missing values. In the DA group, there were four missing values preoperatively, two missing values on day one, and five missing values on day two. In the SPAIRE group there were four missing values preoperatively, two missing values on day one, and three missing values on day two. CRP tests were complete in 99 patients; one patient in the DA group had a missing preoperative CRP value (Figure 1). No patients were lost to follow-up. Baseline data are shown in Table I. Duration of surgery was significantly shorter in the SPAIRE group, 53 minutes (SD 7) compared with 61 minutes (SD 11) in the DA group (p < 0.001).

### CK

CK increased on days one and two post-surgery in both groups to three times the level at baseline: 396 (SD 263) in the DA group compared with 330 (SD 202) in the SPAIRE group. The difference was statistically insignificant (Table II).

**Table II.** Mean values and differences in creatine kinase, CRP, and haemoglobin.

Variable	DA	SPAIRE	Mean difference (95% CI)	p-value*
**CK (U/l)**				
Mean preoperative (SD)	116 (98)	127 (154)	-11 (-62 to 41)	0.690
24 hrs (95% CI)	367 (291 to 427)	322 (269 to 380)	45 (-22 to 151)	0.290
48 hrs (95% CI)	396 (303 to 467)	330 (268 to 400)	66 (-42 to 185)	0.194
**CRP (mg/l)**				
Mean preoperative (SD)	14 (26)	8 (16)	6 (-6 to 10)	0.594
48 hrs (95% CI)	93 (77 to 104)	90 (73 to 106)	3 (-23 to 19)	0.933
**Hb (g/dl)**				
Mean preoperative (SD)	12.9 (1.5)	12.8 (1.4)	0.1 (-0.5 to 0.6)	0.810
48 hrs (95% CI)	11.6 (11.2 to 12)	11.3 (10.9 to 11.7)	0.3 (-0.2 to 0.5)	0.388

* Independent-samples *t*-test.

CK, creatine kinase; DA, direct anterior; Hb, haemoglobin; SPAIRE, sparing piriformis and obturator internus, repair externus.

### CRP

There was an insignificant difference in preoperative CRP levels: 14 mg/l (SD 26) in the DA group compared with 8 mg/l (SD 16) in the SPAIRE group. CRP increased to 91 mg/l (SD 49) in the DA group compared with 90 mg/l (SD 57) in the SPAIRE group on day two after surgery, but the difference was not statistically significant (Table II).

### Hb

Six patients in this study received a blood transfusion after surgery. Four patients in the DA group received a total of 12 units of blood (300 ml per unit). In the SPAIRE group, two patients were transfused with a total of eight units. There was no difference in preoperative Hb values between the groups: 12.9 g/dl (SD 1.5) in the DA group and 12.8 g/dl (SD 1.4) in the SPAIRE group. Hb levels on day two after surgery were lower in both groups but the difference was not statistically significant (Table II).

### Clinical outcomes

Preoperative HHS was 88 (SD 13) in the DA group and 90 (SD 12) in the SPAIRE group. Mean HHS was 83 (SD 15) in the DA group and 80 (SD 16) in the SPAIRE group at three months (95% CI -3.4 to 9.6, p = 0.350). Timed Up and Go (TUG) test^35^ measured on day three was 39.3 seconds (SD 30) in DA and 39.2 seconds (SD 41) in the SPAIRE group. At three months, there was a mean difference of five seconds (13 vs 18) in favour of the DA approach (95% CI -14 to 3, p = 0.224).

A Pearson correlation test revealed non-significant correlations between CK and continuous clinical outcomes (TUG test, HHS, and VAS pain). There was a weak, but statistically significant, negative correlation between CRP on day two and HHS at three months (Table III). Surgical time differed between the groups (53 minutes (SD 7)) in the SPAIRE group compared with 61 minutes (SD 11) in the DA group (p < 0.001). No correlation was found with levels of CK or CRP after adjusting for surgery time.

**Table III.** Pearson correlation coefficients (95% CI) of creatine kinase (CK) and CRP and continuous outcomes.

Score	CK 24 hours	CK 48 hours	CRP 48 hours
TUG 72 hrs	-0.05 (-0.25 to 0.17), p = 0.678	0.55 (-0.16 to 0.27), p = 0.617	0.12 (-0.09 to 0.31), p = 0.256
TUG 3 mnths	-0.03 (-0.25 to 0.19), p = 0.776	-0.26 (-0.25 to 0.19), p = 0.816	0.15 (-0.06 to 0.35), p = 0.151
HHS preop	-0.02 (-0.22 to 0.19), p = 0.869	-0.08 (-0.29 to 0.13), p = 0.464	-0.24 (-0.42 to -0.42), p = 0.018
HHS 3 mnths	0.03 (-0.21 to 0.22), p = 0.978	-0.11 (-0.32 to 0.12), p = 0.351	-0.26 (-0.45 to -0.06), p = 0.012

CK, creatine kinase; HHS, Harris Hip Score; TUG, Timed Up and Go.

### Adverse events

A total of four infections (two in each group), two dislocations (one in each group), and one low-energy periprosthetic fracture (DA approach) was registered during the follow-up period of 12 months. The periprosthetic fracture was revised due to a loose femoral component. One infection was treated with permanent removal of the prosthesis and debridement; three infections were successfully treated with debridement and antibiotics. One dislocation was treated with closed reduction and one was converted to THA with a dual-mobility cup. One patient was admitted with a sacral pressure wound after four months in need of wound debridement.

## Discussion

This study was a pre-planned sub-group analysis from a larger RCT designed to evaluate changes in CK, CRP, and Hb at days one and two postoperatively using the SPAIRE and the DA approach in FNF patients operated with HA. We did not find statistically significant differences between the groups up to 48 hours post-surgery. CK was in both groups within normal levels at admission and was three times the normal value on days one and two after surgery. We found no correlation between CK up to 48 hours postoperatively and clinical outcomes. We found a weak, -0.26, but statistically significant inverse correlation between CRP at admission and HHS at three months. This correlation could be attributed to underlying medical conditions and patients' general medical status.

Results from studies investigating biochemical responses using different surgical approaches diverge. CK and CRP react to muscle trauma and inflammation and are commonly used biomarkers and an objective way to measure the invasiveness of a surgical approach. The SPAIRE approach, being increasingly popular, has to our knowledge not been compared to the DA approach in a RCT.

The DA approach, and its effect on muscle damage and inflammation, has been studied in both osteoarthritis and femoral neck fracture patients with diverging results. Mjaaland et al^25^ reported increased CK values in patients operated with the DA approach compared to the DL approach in THA due to osteoarthritis. They found significant differences in CRP only on day four. The mean difference was 3.1 mg/l. Hoseth et al,^27^ in contrast, reported higher CK values in the DL approach compared to the DA approach in THA due to femoral neck fractures. The difference in CK was only statistically significant on day one after surgery. No significant differences were found on days two, three, and four. They did not find significant differences in CRP on days one or two, but there was higher CRP values in the DL group on days three and four.^27^ The authors suspected that the difference in their results compared with those from Mjaaland et al^25^ may be related to different study populations and differences in preoperative muscle mass in osteoarthritis and FNF patients.

Ugland et al^26^ found elevated levels of CK in the mini-invasive AL group compared to the DL group in HA patients operated due to dislocated FNF. They did not find any correlation between postoperative levels of CK and functional outcomes, such as HHS and TUG test. In a non-randomized study comparing DA and PA in THA, Bergin et al^33^ reported significantly higher CK values in patients operated with PA. Rykov et al^36^ did, however, not find differences in CK or CRP in a study comparing the DA and the PA in THA. Based on the study by Bergin et al,^33^ one would suspect that the SPAIRE approach, a modified posterior approach, resulted in increased CK values compared to the DA approach. This was not found in our study. A possible explanation could be that the SPAIRE is less invasive than the traditional PA. The piriformis and obturator internus muscles are left in situ, leaving musculus obturator externus, together with parts of musculus quadratus, the only muscles detached. Homan retractors facilitate the approach in a similar fashion as in the DA approach.

The low correlation between CK and clinical outcomes in this study could also be attributed to the relatively low increase in CK/CRP. Breakdown of muscle fibres, caused by for example surgery or physical exercise, can be monitored by analysing CK values. Mean CK levels at 24 and 48 hours were 322 U/l and 396 u/l in this study. Exercise-induced rhabdomyolysis causing muscle soreness and pain typically reveals CK levels > 10,000 u/l.^37^ These levels of CK would probably reveal correlation with clinical outcomes. Variations in normal CK values due to age, race, and muscle mass is also well known. Mougios,^38^ investigating CK values in athletes, reported intervals to be 82 u/l to 1083 u/l in male and 47 u/l to 513 u/l in female athletes. The upper reference limits were twice the limits reported for moderately active non‐athletes and up to six times higher than the limits reported for inactive individuals in the literature. Based on this study SPAIRE and DA approaches to the hip joint appear to affect CK and CRP, biomarkers of muscle damage and inflammation, equally in FNF patients operated with HA. No correlation was found to clinical outcomes. However, our baseline CK/CRP values are post-fracture and analyzed at admission to hospital. Muscle mass, age, sex, and time from fracture to admission are factors that could affect baseline figures, and these limitations should be accounted for when interpreting the results.

The ability to mobilize patients early postoperatively is multifactorial and the choice of surgical approach may be a contributing factor. However, CK and CRP outcomes should be used with caution as an argument in favour of specific approaches. Further studies are warranted to establish a possible relationship between the SPAIRE approach and clinical outcomes improving early mobilization of FNF patients.

**Take home message**

- This study revealed no differences in biomarkers for muscle damage on days one and two after surgery with an anterior or a sparing piriformis and obturator internus, repairing externusm (SPAIRE) approach.

- Pearson correlation test revealed non-significant correlations between creatine kinase and continous clinical outcomes.

## Data Availability

The data that support the findings for this study are available to other researchers from the corresponding author upon reasonable request.
